# Pathway Analysis of Smoking Quantity in Multiple GWAS Identifies Cholinergic and Sensory Pathways

**DOI:** 10.1371/journal.pone.0050913

**Published:** 2012-12-05

**Authors:** Oscar Harari, Jen-Chyong Wang, Kathleen Bucholz, Howard J. Edenberg, Andrew Heath, Nicholas G. Martin, Michele L. Pergadia, Grant Montgomery, Andrew Schrage, Laura J. Bierut, Pamela F. Madden, Alison M. Goate

**Affiliations:** 1 Department of Psychiatry, Washington University School of Medicine, St. Louis, Missouri, United States of America; 2 Department of Biochemistry and Molecular Biology, School of Medicine, Indiana University, Indianapolis, Indiana, United States of America; 3 Genetic Epidemiology, Queensland Institute of Medical Research, Brisbane, Australia; 4 Molecular Epidemology, Queensland Institute of Medical Research, Brisbane, Australia; Universidad Europea de Madrid, Spain

## Abstract

Cigarette smoking is a common addiction that increases the risk for many diseases, including lung cancer and chronic obstructive pulmonary disease. Genome-wide association studies (GWAS) have successfully identified and validated several susceptibility loci for nicotine consumption and dependence. However, the trait variance explained by these genes is only a small fraction of the estimated genetic risk. Pathway analysis complements single marker methods by including biological knowledge into the evaluation of GWAS, under the assumption that causal variants lie in functionally related genes, enabling the evaluation of a broad range of signals. Our approach to the identification of pathways enriched for multiple genes associated with smoking quantity includes the analysis of two studies and the replication of common findings in a third dataset. This study identified pathways for the cholinergic receptors, which included SNPs known to be genome-wide significant; as well as novel pathways, such as genes involved in the sensory perception of smell, that do not contain any single SNP that achieves that stringent threshold.

## Introduction

Cigarette smoking is a common habit that has detrimental effects on physical health including an increased risk of heart disease, cancer, stroke, and chronic lung disease. In the United States, cigarette smoking is the leading cause of morbidity and mortality; accounting for 30% of all cancer deaths and 80% of deaths from chronic obstructive pulmonary disease [1]. Although tobacco smoking is a complex multidimensional behavior, research has highlighted smoking quantity, usually evaluated by cigarettes per day (CPD), as a predictor of nicotine dependence [2].

Epidemiological studies have demonstrated that both environmental and genetic factors are associated with different dimensions of smoking behavior [3]. The heritability of smoking quantity is estimated to be between 0.49 and 0.56 [4], and different GWAS have identified and replicated signals within the nicotinic acetylcholine receptor genes on chromosome 15q25 (*CHRNA5-A3-B4*) [5]–[10] and chromosome 8p11 (*CHRNB3-A6*) [8], [9], as well as nicotine metabolizing genes on chromosome 19q13 (*CYP2A6-B6*) [9], [10];. Each of these variants explains 0.5∼1.9% of the phenotypic variance for subjects of European ancestry (EA) [10], [11]. Additional meta-analysis efforts [7] have been carried out to identify more loci, presumably with smaller effect and only detectable by the power gained by a larger number of samples. The most encouraging signals found in a discovery phase, that included more than 15,000 subjects with reported values for smoking quantity, were followed up in a replication phase which encompassed two studies that gathered even larger pools of subjects (ENGAGE [9] *n* = 46,481; and TAG [10] *n* = 74,035). However, no additional locus achieved genome-wide significance (p-value<5.0E−8 for 1 million SNPs tested), raising the possibility that structural or rare variants with strong effects explain the missing heritability [12].

That only a small fraction of the heritability of smoking quantity is detected by GWAS is a characteristic common to many other complex traits [13]. Preliminary estimates of the total amount of phenotypic variance explained by common SNPs (minor allele frequency >1%) [13], suggests that similar to other complex traits [13], [14], the percentage of genetic variance captured by GWAS chips is higher than that explained by the loci identified so far; this suggests that many of the signals that fail to reach genome-wide significance in current datasets are true signals.

Pathway analysis offers a complementary perspective to interpret GWAS, incorporating repositories of expert knowledge, represented in biological pathway databases and gene ontologies. This approach evaluates whether the signals detected by a GWAS are overrepresented for families of biologically related genes. By shifting from the evaluation of individual SNPs to pathways of genes –under the commonly accepted hypothesis that causal variants are not randomly distributed across the genome, but instead lie in functionally related genes– we can prioritize the variants that do not reach genome-wide significance level [15]. This concept was extensively employed in the identification of expression profiles of microarrays, and since then has been adapted to mine GWAS datasets [15]–[17]. Employing different statistical methods and implementations, pathway analysis has been applied to a variety of neurological and psychiatric diseases [17]; implicating axon guidance for Parkinson disease [18], neuronal cell adhesion and membrane scaffolding for schizophrenia and Bipolar disorder [19], and immune system and cholesterol metabolism for Alzheimer’s disease [20].

We applied pathway analysis to two substance dependence GWAS datasets, the “Nicotine addiction Genetics” (OZALC-NAG) [21], and the “Study of Addiction: Genetics and Environment” (SAGE) [22], analyzing a commonly used measure of smoking behavior [7], [9], [10], cigarettes per day (CPD )(Table 1), to identify enriched candidate sets of genes defined as Gene Ontology (GO) terms [23] and Kyoto Encyclopedia of Genes and Genomes (KEGG) pathways [24]. We carried out the statistical analysis by executing the algorithm ALIGATOR [15] an overrepresentation method that analyzes genes exhibiting significance below a specified threshold. We analyzed these two studies independently, and selected those terms and pathways that were statistically significant (*p-value*<0.05) in both datasets, and further verified their role by analyzing the EA subjects from the Atherosclerosis Risk in Communities study (ARIC) [25], [26]. We applied this same approach by executing the algorithm MAGENTA [27] an implementation of the method Gene Set Enrichment Analysis (GSEA).

**Table 1:** Characteristics of the studies and subjects analyzed.

		OZALC-NAG	SAGE	ARIC
# Subjects		4038	2014	5198
% Men/%Women		49%/51%	44%/56%	56%/44%
Age, mean ± S.D.		45.16±10.43	37.97±9.09	54.30±5.7
Cigarettes per day				
	*0 to 10*	1159	1001	1047
	*11 to 20*	876	518	2155
	*21 to 30*	958	223	941
	*30 or more*	1045	272	1052

* CPD: Cigarettes per day.

## Materials and Methods

### Samples and Study Design

We analyzed the subjects with European ancestry with reported cigarettes per day (CPD) values included in the Nicotine Addiction Genetics (OZALC-NAG) study [28]; the Study of Addiction: Genetics and Environment (SAGE) [22]; and the Atherosclerosis Risk in Communities study (ARIC) [25], [26] (Table 1). Both the OZALC-NAG, which is an Australian family based study, and the SAGE study, which includes unrelated North American subjects, are GWAS ascertained based on substance dependence. In contrast ARIC is a population-based study designed to investigate the etiology of atherosclerosis in middle-aged adults [25]. Additional details for each of these studies is provided in Text S1 and elsewhere [28], [22] and [26].

### Genotypes

We implemented a unifying strategy that employed both genotyped and imputed SNPs to analyze the same set of SNPs in each of the three studies (Text S2). We analyzed all of the SNPs included in the Illumina Human 1 M beadchip to maximize the signals ascertained in the two exploratory studies.

### Pathway Analysis

#### ALIGATOR [15]

This method performs an overrepresentation analysis, evaluating the significance for each category of genes empirically. This method can be applied to both unrelated and family based datasets. It selects the set of genes, of size *n,* which are tagged by SNPs located within gene sequences or in the 20 kb up/downstream flanking these gene regions, which are more significant than a specific threshold (i.e., <0.001; 0.005; 0.01; and 0.05). The association p-value is estimated using standard GWAS methods, and is detailed in Text S3. A pruning process that eliminates SNPs in linkage disequilibrium is performed by considering only the most significant SNP among all of the SNPs that have r^2^>0.2 and are within 1 Mb. If one SNP tags more than one gene, all of these genes are included as significant. Although more than one SNP in linkage equilibrium (r^2^<0.2) might tag a gene, each gene is counted only once. The statistical significance of the overrepresentation of each set of genes (category-specific *p-value*) is calculated by comparing the number of significant genes to the number of genes expected by chance. For this purpose, the algorithm generates 50,000 sets of genes, by randomly selecting SNPs until a list of *n* tagged genes is formed.

Random sets of genes are also employed to estimate the study wide *p-value* by applying a bootstrap method: one of the 50,000 sets of genes is selected as a reference and compared to a subsample of 5,000 random lists (selection with replacement). This is repeated 1,000 times, comparing the most significant *p-value* in each iteration to the *p-value* of each category of genes, estimating how often the significance level is obtained by random chance. In addition, the number of sets of genes that achieved different threshold levels (i.e., *<*0.005; 0.01 and 0.05) is compared to the values obtained by random reference studies to calculate the excess of significantly overrepresented sets of genes [15]. The method can correct for any bias due to genes that are physically located within the same region of the chromosome (genes which are less than 1 Mb apart) associated with the same signal and assigned to the same category of genes. This is done by grouping these genes as a single entity, which is tagged by a set of SNPs mapping within the genes.

#### MAGENTA [27]

This method implements a gene set enrichment analysis [29] (GSEA) without requiring the empirical phenotype-based test procedure to estimate the significance of the categories of genes, enabling its application to family based studies as well as population-based genome-wide association study meta-analyses. In our work. each gene is scored by the most significant *p-value* among all of the SNPs located within the gene or up to 20 kb from the 5′ and 3′ ends of the genic sequences (Text S2). This value is corrected for confounding effects: the gene size, number of SNPs per kb, number of independent SNPs per kb, number of recombination hotspots per kb, linkage disequilibrium units per kb, and genetic distance measured in centiMorgans per kb. This is done by applying a step-wise multiple linear regression analysis to the normalized (Z-score) *p-value* of each gene [27]. MAGENTA also corrects for physical proximity along the chromosome retaining only one gene per cluster of genes in a category of genes. The nominal GSEA *p-value* is calculated by comparing the extent of the “leading edge fraction” (i.e., the subset of genes whose corrected *p-values* are among the 95^th^ percentile) of each category of genes (of size *n*), to the one observed in 10,000 random samples of *n* genes. Finally, the method uses Bonferroni multiple test correction (*p-value* = 0.05), and also computes the false discovery rate, a less stringent approach to correct for the burden of testing multiple hypotheses.

### Enrichment of Categories of Genes Common to OZALC-NAG and SAGE Studies

The statistical significance of the number of categories of genes enriched in both the OZALC-NAG and SAGE studies was empirically evaluated by executing ALIGATOR to evaluate the OZALC-NAG study on the subset of categories of genes significantly enriched in the SAGE study.

### Biological Repositories of Expert Knowledge Analyzed

For the current analysis we included Gene Ontology terms [23] (April 2010) and Kyoto Encyclopedia of Genes and Genomes pathways [24] (October 2010) that contain between 3 and 500 genes interrogated by the Illumina 1 M platform. This resulted in a total of 7962 GO terms and 217 KEGG pathways that we refer to as categories of genes.

## Results

### Approach to Identify GO Terms and KEGG Pathways Affecting Cigarette Consumption

We applied the ALIGATOR method independently to the OZALC-NAG study and the EA subjects from the SAGE study; analyzing the subset of SNPs that reached different significance levels (i.e., SNP *p-value*<0.001; 0.005; 0.01; and 0.05), to circumvent any bias introduced by this parameter [15]. We observed that for every combination of SNP *p-value* and category of gene enrichment *p-value* thresholds, there were a significant number of categories of genes enriched in common in the OZALC-NAG and SAGE studies (*p-value*<0.01) (Table 2). We selected the thresholds for the SNP and category of gene *p-values* (Table 2) that identified a significant excess of enriched categories of genes (*p-value*<0.05) for both studies. (See Table S1 panels A and B to see specific Gene Ontology and KEGG results respectively). We discarded those values for the SNP and category of gene threshold that were significant for only a single study: for example, although the OZALC-NAG study show a significant excess of pathways (*p-value* = 0.0346) with *p-values* lower than 0.005 when we analyzed the SNPs with *p-values* lower than 0.005, the SAGE study was not significant (*p-value* = 0.141) for these same cutoff values (Table 2). Because of this lack of consistency, we did not consider the categories of genes identified by these specific values for the thresholds. In contrast, there was a replicated significant excess of categories of genes for both studies when the SNPs with *p-values* <0.001 were analyzed. This occurred independently of the threshold for categories considered (i.e., 0.005, 0.01 and 0.05) (Table 2); thus we considered all of the terms and pathways with a category-specific *p-value* that satisfied the most relaxed constraint for category cutoff (i.e.,  = 0.05), which includes all of the pathways satisfying the more stringent constraints. When we analyzed the SNPs with *p-values* <0.05 we observed a significant excess of enriched categories of genes with *p-values* <0.005 and 0.01 consistently in both OZALC-NAG and SAGE studies (Table 2). Applying the same criteria described above, we analyzed the categories with category-specific *p-values* <0.01. Our replication strategy was to evaluate the subset of categories of genes that were significant for both the OZALC-NAG and the SAGE studies in the ARIC study (*p-value*<0.05) (See Table 2 for the number of common categories of genes between the two studies and its statistical significance).

**Table 2:** Number of categories of genes identified by ALIGATOR for OZALC-NAG and SAGE studies for smoking quantity.

				Category of Gene enrichment threshold					
				0.005		0.01		0.05	
SNP Threshold	Study	# SNPs	# Genes	#cat.	*p-value*	#cat.	*p-value*	#cat.	*p-value*
0.001	OZALC-NAG	270	264	24	1.40E−03	36	3.80E−03	102	1.32E−02
	SAGE	206	225	15	7.60E−03	35	2.40E−03	122	1.60E−03
	Common			4	<2.00E−4	5	<2.00E−4	7	8.20E−03
0.005	OZALC-NAG	1158	1078	18	3.46E−02	27	1.04E−01	147	7.94E−02
	SAGE	1094	1127	11	1.14E−01	35	5.40E−02	181	2.92E−02
	Common			2	<2.00E−4	2	<2.00E−4	14	1.20E−03
0.01	OZALC-NAG	2242	1962	19	6.18E−02	37	1.06E−01	175	1.41E−01
	SAGE	2150	2021	21	4.42E−02	41	7.72E−02	197	8.16E−02
	Common			2	<2.00E−4	4	<2.00E−4	12	1.60E−03
0.05	OZALC-NAG	10374	6832	39	2.62E−02	72	4.32E−02	281	1.70E−01
	SAGE	9906	6901	46	1.06E−02	85	1.88E−02	326	6.70E−02
	Common			9	<2.00E−4	12	<2.00E−4	55	<2.00E−4

# **SNPs**: number of SNPs that achieved a significant for each threshold for OZALC-NAG and SAGE studies;

# **Genes**: number of genes mapped;

# **cat** and ***p-value reflect*** the excess of categories of genes (GO terms or KEGG pathways) for the thresholds applied to the category-specific *p-values* in the OZALC-NAG and the SAGE studies. “Common” rows show the number of overlapping categories of genes significantly enriched for both studies and the corresponding statistical significance.

For the most stringent threshold for SNPs (*p-value<*0.001) ALIGATOR identified 102 and 122 categories of genes in OZALC-NAG and SAGE respectively (category-specific *p-value<*0.05)(Table 2). Seven GO terms were significant for both studies (Table S2) but no significant KEGG pathways were common to both studies. Moreover, all seven GO terms were also significant for the ARIC study (*p-value*<0.05) (Table S2). These terms group genes in the cluster of cholinergic nicotinic receptor genes on chromosomes 15 and 8, and closely related genes.

We also inspected the categories of genes identified by the most relaxed threshold for SNPs (i.e., *<*0.05). A total of 72 and 85 categories were significant for OZALC-NAG and SAGE respectively (category-specific *p-value* <0.01) (Table 2). Eleven GO terms and one KEGG pathway were common to both studies. These terms and pathways showed an overlap among the constituent genes; thus we grouped these into 4 clusters of categories of genes that reflected the similarity of the genes included (Table 3; Figure S1). These clusters were labeled *i*) cholinergic nicotinic receptors, *ii*) sensory perception of chemical stimulus/smell related genes, *iii*) ribosome genes, and *iv*) retinoid binding genes. Eight of these categories of genes were significant in ARIC (category-specific *p-value*<0.05). Neither of the two retinoid binding GO terms showed statistical significance for the ARIC study. In contrast, each of the remaining 3 clusters included at least one GO term with category-specific *p-values* lower than 0.01 for the ARIC study.

**Table 3:** ALIGATOR identified common OZALC-NAG and SAGE GO terms and KEGG pathways for the analysis of SNPs p-value<0.05 and ARIC replication results.

				OZALC-NAG			SAGE			ARIC			Combined
acc		name	# Genes Cat.	# Genes	*p-value*	Expected	# Genes	*p-value*	Expected	# Genes	*p-value*	Expected	*p-value* *
Cholinergic nicotinic receptors													
	GO:0042166	acetylcholine binding	22	11	5.80E−04	3.30	10	2.32E−03	3.3348	8	7.10E−03	2.69	2.57E−06
	GO:0015464	acetylcholine receptor activity	18	10	9.00E−04	2.86	8	9.98E−03	2.8908	7	1.12E−02	2.34	1.17E−05
	GO:0004889	nicotinic acetylcholine-activated cation-selective channel activity	17	9	4.40E−03	3.05	10	1.20E−03	3.0838	7	1.50E−02	2.49	2.40E−05
	GO:0005892	nicotinic acetylcholine-gatedreceptor-channel complex	16	9	4.40E−03	3.05	10	1.20E−03	3.0838	7	1.50E−02	2.49	1.24E−04
Sensory perception													
	GO:0004984	olfactory receptor activity	369	82	4.00E−05	48.48	78	1.00E−04	48.9594	61	1.08E−03	39.61	8.11E−09
	GO:0007608	sensory perception of smell	386	91	9.40E−04	64.19	84	9.16E−03	64.8288	66	3.71E−02	52.42	5.44E−05
	GO:0007606	sensory perception of chemical stimulus	430	99	8.00E−04	70.76	91	9.06E−03	71.4626	70	*5.20E*−*02*	57.77	7.70E−05
Ribosome													
	hsa03010	Ribosome	88	15	3.40E−04	5.36	19	2.00E−04	5.4116	14	1.60E−04	4.37	1.13E−08
	GO:0005840	ribosome	192	34	5.00E−04	17.97	40	2.00E−04	18.1458	32	8.00E−04	14.67	5.69E−08
	GO:0022625	cytosolic large ribosomal subunit	36	8	4.82E−03	2.53	9	1.52E−03	2.557	5	*6.17E*−*02*	2.05	1.73E−04
Retinoid													
	GO:0005501	retinoid binding	26	9	2.36E−03	5.54	9	2.60E−03	4.0218	6	*1.71E*−*01*	4.84	5.53E−04
	GO:0001972	retinoic acid binding	11	3	1.82E−03	0.68	4	6.40E−04	1.0174	1	*5.82E*−*01*	3.00	2.80E−03

**acc:** Category of gene (prefix GO indicates Gene ontology and hsa KEGG pathways) and corresponding **name**;

# **Genes Cat.:** the total number of genes grouped by the category. For each study:

# **genes** significant; corresponding category-specific ***p-value***; and the **Expected** number of genes.

* Combined p-values were calculated employing the weighted Z-score method.

### GO Terms Enriched for Cholinergic Receptor Genes

**Figure 1:**
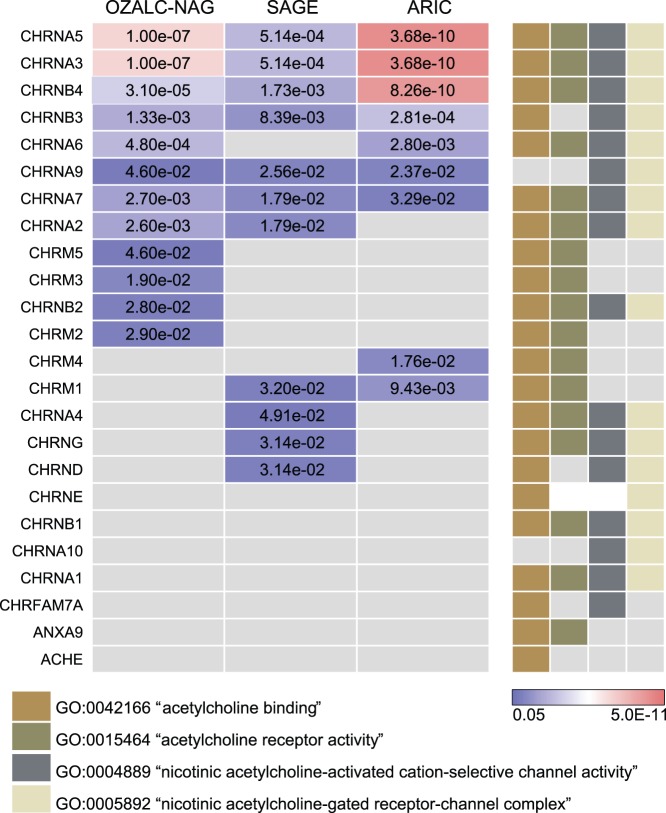
Go terms for cholinergic receptors and significant genes. The p-value of each gene was assigned based on the most significant SNP in gene sequences and flanking regions (Left panel). SNPs in linkage disequilibrium (r^2^>0.2) and in a local proximity (1 Mb) were removed. Colored boxes in the right panel reflect the assignment of each gene to the different GO terms.

We identified terms for cholinergic nicotinic receptor genes (*CHRN*) that were significant independent of the threshold used to select the SNPs. Three molecular function and one cellular component terms were significant for both OZALC-NAG and SAGE, regardless of the threshold for SNPs considered (Table 3 and Table S2). These four terms were replicated in ARIC, again for both *p-value* thresholds applied to SNP selection. Despite specificity differences in the genes grouped by each term, all of them contain a majority of the nicotinic (*CHRN*) and muscarinic (CHRM) cholinergic receptor subunit genes, and other closely related genes (Figure 1).

All of these terms included the clusters of cholinergic receptors on chromosome 15 (*CHRNA5-A3-B4*) [5]–[10] and chromosome 8 (*CHRNB3-A6*) [8]–[10]. By virtue of the broad range of SNP significance analyzed, these genes were tagged in each of the 3 studies, whether or not the significant SNPs achieved genome-wide level (Figure 1; See Table S3 for the rs numbers of the SNPs tagging the genes in cholinergic receptor genes). In addition, several other cholinergic receptors were tagged by SNPs with more moderate *p-values* (<0.05) (Figure 1). *CHRNA7* (chr. 15q14) [11] and *CHRNA9* (chr. 4p14) were tagged in each of the three studies (Figure 1), suggesting that variants in these genes, are also implicated in the genetic susceptibility to smoking quantity. To determine whether the nicotinic receptor genes on chromosomes 15 and 8 drove the statistical significance of these terms we analyzed a reduced dataset that did not include these genes (*i.e.*, we removed *CHRNA5-A3-B4* and *CHRNB3-A6* genes). Although there was a considerable difference in the significance, the terms GO:0005892, GO:0042166 and GO:0004889 showed *p-values* <0.05 for SAGE (*i.e.*, 0.014, 0.02 and 0.014 respectively) and a marginal *p-value* of 0.056 for the term GO:0015464. In the OZALC-NAG study, the term GO:0015464 showed a *p-value* of 0.049, and the term GO:0042166 a marginal *p-value* of 0.06. None of these terms was significant in ARIC, which reported the fewest significant genes for these terms (Table 3; and Figure 1), and only included 4 genes (*CHRNA7/9* and *CHRM4/1*) after dropping the genome-wide significant genes.

The MAGENTA algorithm only identified GO terms related to cholinergic nicotinic genes (See Table 4 for significance results, and Table S4 for false discovery rates). Seven of the terms identified for OZALC-NAG and SAGE were also significant in ARIC (nominal *p-value*<0.05). Moreover, three of these terms were previously detected by ALIGATOR (*i.e.,* GO:0015464, GO:0005892 and GO:0004889). In contrast to the other terms detected by MAGENTA, the term GO:0005230, ancestor of GO:0005230, includes genes other than the cholinergic receptors. However, none of these seven terms remained significant in any study when we performed the analysis dropping the clusters of cholinergic genes located in chromosomes 15 and 8, indicating that this core of cholinergic nicotinic receptors drives the results observed using MAGENTA.

**Table 4:** MAGENTA Significant categories of genes with nominal p-value<0.05 in the OZALC-NAG and the SAGE studies and ARIC corresponding results.

			OZALC-NAG	SAGE	ARIC	Combined
Threshold	Acc	Name	*p-value*	*p-value*	*p-value*	*p-value* *
0.005	GO:0035095	behavioral response to nicotine	2.20E−03	2.20E−03	2.00E−03	8.29E−06
	GO:0060084	synaptic transmission involved in micturition	8.50E−03	7.20E−03	7.90E−03	1.91E−04
						
0.01	GO:0006942	regulation of striated muscle contraction	6.50E−03	6.30E−03	1.00E+00	2.94E−01
						
0.05	GO:0004889	nicotinic acetylcholine-activated cation-selective channel activity	8.50E−05	4.06E−02	6.00E−04	7.83E−07
	GO:0005892	nicotinic acetylcholine-gated receptor-channel complex	8.20E−05	4.36E−02	1.10E−03	1.60E−06
	GO:0015464	acetylcholine receptor activity	2.68E−04	2.50E−02	2.20E−03	5.90E−06
	GO:0005230	extracellular ligand-gated ion channel activity	3.50E−03	1.65E−02	1.76E−02	3.43E−04
	GO:0007271	synaptic transmission, cholinergic	3.30E−03	2.63E−02	2.60E−02	6.41E−04
	GO:0042060	wound healing	3.00E−04	1.69E−02	3.22E−01	6.05E−03
	GO:0006940	regulation of smooth muscle contraction	2.50E−02	2.61E−02	1.33E−01	1.68E−02
	GO:0005216	ion channel activity	2.52E−02	1.53E−02	1.88E−01	2.23E−02
	GO:0042552	myelination	1.51E−02	1.64E−02	4.35E−01	5.94E−02
	GO:0007257	activation of JUN kinase activity	3.42E−02	3.61E−02	3.52E−01	7.47E−02
	GO:0006548	histidine catabolic process	4.71E−02	4.27E−02	3.04E−01	7.52E−02
	GO:0034185	apolipoprotein binding	4.20E−02	4.12E−02	1.00E+00	1.45E−01
	GO:0007265	Ras protein signal transduction	4.17E−02	4.99E−02	8.33E−01	3.14E−01

**Threshold** of significance satisfied by both NAF and SAGE; **acc**: identifier for the category of genes and name. For each study, the nominal GSEA *p-value* is shown (see Table S4 for false discovery rate).

* Combined *p-values* were calculated employing the weighted Z-score method.

### Sensory Perception of Chemical Stimulus and Smell

The most abundant categories of genes, with regard to the number of genes included, that ALIGATOR identified in the OZALC-NAG and SAGE studies grouped highly similar sets of genes. The biological processes GO:0007608, its ancestor the term GO:0007606 and the molecular function term GO:0004984 shared 368 genes in common (Table 3). From the pool of 431 different genes grouped by these terms, 56 were significant for at least two studies (Figure S2), and 7 genes were common to all three studies. This list includes the glutamate receptors, metabotropic 7 (*GRM7*– chr. 3p26.1-p25.1) and 8 (*GRM8*– chr. 7q31.3-q32.1); the olfactory receptors *OR10P1* (chr. 12q13.2); *OR52E2* (chr. 11p15.4), *OR52J3* (chr. 11p15.4), and *OR8D4* (chr. 11q24.1); and the bitter taste receptor *TAS2R1* (chr. 5p15).

Both *GRM7* and *GRM8* are part of the glutamate signaling pathway and were previously reported to be associated with nicotine dependence [30], [31] and smoking initiation [32] respectively. In addition, a genetic linkage peak near GRM7 and nominal association was reported for GRM7 and major depression in the OZALC-NAG heavy smoking families [33]. rs963843 (MAF = 0.14) in GRM8 was the most significant SNP for SAGE (p-value = 5.0E−3), with an increased number of CPD. Although not significant, we observed the same direction of effect for this SNP in both OZALC-NAG (*p-value* = 0.074) and ARIC (*p-value* = 0.10), with the combined *p-value* = 1.16E−3. Two additional SNPs, the protective rs1557644 (p-value = 3.0E−3; MAF = 0.35) and the risk rs1018854 (*p-value* = 3.27E−4; MAF = 0.44) were significant in OZALC-NAG and ARIC respectively. Moreover, the haplotype derived from the risk alleles showed a p-value = 5.40E−3 (effect = 0.21) for SAGE.

### Retinoid Binding Genes

Two molecular function GO terms significant for both the OZALC-NAG and SAGE studies group genes related to retinoid binding (Table 3), and exhibit a hierarchical relationship among them, GO:0001972 being the most specific and the term GO:0005501 the most general. Both studies share 4 significant genes (Figure S3) encoding the cellular retinoic acid binding proteins 1 (*CRABP1* - chr 15q24), and 2 (CRABP2 - chr. 1q21.3); insulin-like growth (*IGF2R* - chr 6q26); and the complex of genes *UDP* glucuronosyltransferase 1 family, polypeptide A (UGT1A1/3/4/6/7/8/9/10), which are associated with the metabolism of nicotine [34]. Due to the physical overlap on the chromosome of this complex of genes, we employed *UGT1A4*, as a representative of the entire complex, eliminating the other members from the database of terms.

None of these terms was significant for ARIC, though the term GO:0016918 “*retinal binding*”, which represents a subset of the term GO:0005501 was significant (*p-value* = 0.045). This term also includes the gene *CRABP1* (Figure S3), which was significant for ARIC. Among the other genes in the term GO:0016918 in ARIC we found 3 common to OZALC-NAG (Figure S3). A posterior inspection of the ARIC study showed that the gene *IGF2R*, also had significant SNPs (i.e., rs8191772 with a p-value = 0.01). In contrast, no significant SNP tagged either *CRABP2* nor *UGT1A4*.

## Discussion

Our approach to analyzing susceptibility pathways for smoking quantity was based on the identification of GO terms and KEGG pathways common to the OZALC-NAG and SAGE datasets that showed replication in the ARIC study. Our decision to unify the set of SNPs analyzed in each of the two exploratory studies, genotyped by different Illumina chips, proved to be a valid option that identifies common categories of genes while maximizing the chances of observing the same enriching genes. We implemented this strategy by extending the set of SNPs originally genotyped in the OZALC-NAG study, incorporating imputed data to encompass the ones ascertained in SAGE. We applied this same approach to analyze the ARIC dataset, which was genotyped using the Affymetrix platform. Moreover, apart from the differences in the enriching genes, all of the replicated GO terms and KEGG pathways were also significant when we restricted analysis of ARIC to the genotyped SNPs in the Affymetrix Human SNP Array 6.0 (data not shown).

We found GO terms for the cholinergic receptors, that included genes tagged by SNPs previously reported to achieve genome-wide significant levels (i.e. 5.0E−08), although these SNPs did not necessarily achieve this level in the datasets we evaluated. In addition, other cholinergic receptor genes with more modest *p-values* were also enriched in these GO terms. Each study identified CHRNA7 but the significant SNPs were different and in low r^2^ (<0.20) but high D’ (>0.80) values, suggesting the existence of a shared risk allele. However, we could not identify a same SNP that was significant for the three studies in the region (*p-value*<0.05). This might indicate the presence of an untyped SNP, possibly with a minor allele frequency too low to be accurately imputed, or might be a synthetic association representing the effects of multiple rare variants. In contrast, for the CHRNA9 gene, we could neither identify a common significant SNP nor a common allele tagged by SNPs in linkage disequilibrium (r^2^>0.5 or D’>0.5). Despite this, the pathway analysis was robust enough to highlight the associations of these two genes to smoking quantity. Indeed, the presence of these moderate signals in *CHRNA7* and *CHRNA9*, as well as the ones in cholinergic muscarinic receptors, sustained the significance of the terms GO:0005892, GO:0042166, GO:0004889 and GO:0015464 for the analysis of the least stringent threshold for SNPs (Table 3). In contrast, the absence of these moderate signals resulted in the terms GO:0035095, GO:0035094 and GO:0007274 only being significant for the analysis of the SNPs that satisfied the most stringent threshold (Table S2).

We did not restrict our analysis to the ALIGATOR method, but also applied the MAGENTA method, as each of these methods can provide complementary findings [35]. Only the GO terms for cholinergic receptors were consistently significant in the OZALC-NAG, SAGE and ARIC studies using the MAGENTA method, increasing the levels of certainty of the original ALIGATOR predictions. In contrast, the ALIGATOR method identified other GO terms and KEGG pathways common to the three datasets. We verified if the assumption of one causal gene per signal made by the MAGENTA method caused the different results. However, correcting or not for physically proximal genes did not change substantially the MAGENTA results for these categories of genes. It could be argued that ALIGATOR results are a product of the specificity of the method, and are not susceptibility factors for smoking. However, some of the genes included in these significant GO terms were previously reported to influence smoking behaviors, which increases the confidence in these findings (*e.g., GRM8* [32] and *GRM7* [31], [30]). Our analysis detected other genes common to all datasets, including the bitter taste receptor *TAS2R1*, suspected to be able to sense the nicotine in cigarette smoke [36]. It has been shown that nicotine activates taste receptor pathways both specific for nicotine and also common to other bitter substances [37], [38]. This provides support for the finding that variants in some of the taste receptors can modulate cigarette consumption. Similarly, retinoic acid genes were also specific to the ALIGATOR analysis; but again it has been suggested that the activation of nicotinic receptors affects cellular signaling associated with retinoic acid target genes [39].

One caveat to consider when performing pathway analysis is that the results obtained are biased, or at least restricted, to the biological knowledge that is incorporated into the GWAS, as well as its representation and modeling. This may explain the absence of any replicated GO term or KEGG pathway for the metabolism of nicotine. *CYP2A6* encodes the enzyme that metabolizes approximately 70 to 80% of nicotine to cotinine [10], [40], [41]. A single SNP in this chromosomal region is typed by the Illumina Human 1 M chip.: rs3733829, which is in the first intron of EGLN2 located 40 kb downstream from *CYP2A6* [10]. This SNP is not in high linkage disequilibrium with any other SNP included in the chip in the extended genetic region considered for the *CYP2A6*. Moreover, *CYP2A6* is a complex locus involving structural and rare functional variants that are not well tagged by the SNPs included in the genotyping platforms. The *UGT* complex of genes, which catalyze nicotine and cotinine glucuronidation [34], were significant in both the OZALC-NAG and SAGE studies, and were identified among the other retinoid binding genes. Furthermore, the flavin monooxygenase 1 gene (*FMO1*) is also associated with nicotine metabolism [42] and was tagged in the three studies. There is one KEGG pathway (hsa00982 “*Drug metabolism – cytochrome P450*”) and two GO terms (GO:0005792 “*microsome*” and GO:0042598 “*vesicular fraction*”) with less than 500 genes that include FMO1 and UGT1A4. However, each of these terms and pathways has a very large number of genes (i.e., 73, 241 and 248 genes respectively); and thus are not specific enough to formally represent nicotine metabolism genes. In contrast, the identification of ribosome genes was an unexpected result of our analysis. The relationship, if any, of this gene family to nicotine consumption is not currently understood.

Both ALIGATOR and MAGENTA provide methods to correct for the multiple pathways tested. ALIGATOR applies a bootstrap approach [15] whereas MAGENTA implements both Bonferroni multiple test correction and false discovery rate method [27]. To analyze the combined evidence of the multiple studies evaluated independently (Table 3) we chose the most stringent method, the Bonferroni multiple test correction, although it is most likely too conservative for the nested organization of GO terms. The corrected *p-value* is 6.11E−6 for the nominal *p-value* = 0.05 and 8179 pathways tested. Using this significance level the terms GO:0042166 and GO:0004984 were significant for the three combined studies (weighted Z-method [43]) (Table 3). Similarly, both methods provide mechanisms to correct for linkage disequilibrium; and thus avoid the situation that a same signal, which spans across multiple genes, inflates the number of significant genes in a same pathway. Because this inflation can be a source of false positive results for terms including clusters of genes, we re-executed the ALIGATOR method collapsing all physically proximal genes in the same category into a single entity. The GO terms representing the cholinergic receptor genes remained significant (category-specific *p-value* <0.05) after this correction (Table S5) for the OZALC-NAG, the SAGE and the ARIC studies. Similarly the GO term GO:0004984 *“olfactory receptor activity”* remained significant in the three studies, but the other two sensory perception GO terms were not consistently significant (Table S5).

Our systematic analysis of smoking quantity, conditioning GWAS to extrinsic information, has identified both expected and novel GO terms and KEGG pathways. This new information should lead to a further prioritization of genes that do not include genome-wide significance SNPs. Many genetic variants, each one with small effects, are expected to be associated to complex traits [44], Pathway analysis can be considered as a signal-to-noise filter for the true signals that are not strong enough to clearly stand out from the statistical background in a traditional GWAS.

## Supporting Information

Figure S1 **Hierarchical clustering of identified GO terms and KEGG pathway.** We calculated the similarity matrix among the genes included in the 11 GO terms and KEGG pathway; and created a dendrogram by employing the single linkage method (i.e., nearest-neighbor).
(TIFF)

Figure S2 **GO terms for the sensory perception of chemical stimulus and smell and significant genes.** The p-value of each gene was assigned based on the most significant SNP in gene sequences and flanking regions (Left panel). SNPs in linkage disequilibrium (r2>0.2) and in a local proximity (1 Mb) were removed. Colored boxes in the right panel reflect the assignment of each gene to the different GO terms. Only genes significant in at least two studies are reported.
(TIFF)

Figure S3 **Significant genes for significant GO terms related to Retinoid binding terms.** Similar to Figure S2, the *p-value* of genes significant in any of the three studies (OZALC-NAG, SAGE or ARIC) is reported.
(TIFF)

Table S1 **Excess of enriched categories of genes identified by ALIGATOR for OZALC-NAG and SAGE studies for smoking quantity for Gene Ontoloty terms (A) and KEGG pathways (B).**
(PDF)

Table S2 **ALIGATOR identified common OZALC-NAG and SAGE GO terms for the analysis of SNPS p-value <0.001 and ARIC replication results. acc:** Category of gene and corresponding **name**; **# Genes Cat.:** the total number of genes grouped by the category; **# genes** significant; and category specific ***p-value***; and **Expected** number of genes for SAGE, OZALC-NAG and ARIC studies. *Combined p-values were calculated employing the weighted Z-score method {Stouffer:1949ua}
(PDF)

Table S3 **RefSNP (rs) numbers for the SNPs tagging the GO terms for the cholinergic receptor genes.**
(PDF)

Table S4 **MAGENTA False discovery rate for categories of genes with nominal p-value<0.05 in the OZALC-NAG and SAGE studies and ARIC corresponding results.**
(PDF)

Table S5 **ALIGATOR enrichment analysis of the collapsed genes (physical distance <1** **Mb) for GO terms representing the cholinergic receptor and the sensory perception genes.**
(PDF)

Text S1 **Samples and study design.**
(DOCX)

Text S2 **Genotypes.**
(DOCX)

Text S3 **Statistical analysis.**
(DOCX)
