# Correction: Pathway Analysis of Smoking Quantity in Multiple GWAS Identifies Cholinergic and Sensory Pathways

**DOI:** 10.1371/annotation/21033005-e120-4364-8024-c6534b4108f6

**Published:** 2013-08-05

**Authors:** Oscar Harari, Jen-Chyong Wang, Kathleen Bucholz, Howard J. Edenberg, Andrew Heath, Nicholas G. Martin, Michele L. Pergadia, Grant Montgomery, Andrew Schrage, Laura J. Bierut, Pamela F. Madden, Alison M. Goate

The current funding information is incorrect. The full, correct funding statement can be found below.

OZALC-NAG is supported by National Institutes of Health Grants DA012854, AA07535, AA07728, AA13320, AA13321, AA14041, AA11998, AA17688 and DA019951; by grants from the Australian National Health and Medical Research Council (241944, 339462, 389927, 389875, 389891, 389892, 389938, 442915, 442981,496739, 552485 and 552498); by grants from the Australian Research Council (A7960034, A79906588, A79801419, DP0770096, DP0212016, and DP0343921); and by the 5th Framework Programme (FP-5) GenomEUtwin Project (QLG2-CT-2002-01254). Genome-wide association study genotyping at the Center for Inherited Disease Research was supported by a grant to the late Richard Todd, M.D., Ph.D., former Principal Investigator of grant AA13320. Funding support for the Study of Addiction: Genetics and Environment (SAGE) was provided through the NIH Genes, Environment and Health Initiative [GEI] (U01 HG004422). SAGE is one of the genome-wide association studies funded as part of the Gene Environment Association Studies (GENEVA) under GEI. Assistance with phenotype harmonization and genotype cleaning, as well as with general study coordination, was provided by the GENEVA Coordinating Center (U01 HG004446). Assistance with data cleaning was provided by the National Center for Biotechnology Information. Support for collection of datasets and samples was provided by the Collaborative Study on the Genetics of Alcoholism (COGA; U10 AA008401), the Collaborative Genetic Study of Nicotine Dependence (COGEND; P01 CA089392), and the Family Study of Cocaine Dependence (FSCD; R01 DA013423, R01 DA019963). Funding support for genotyping, which was performed at the Johns Hopkins University Center for Inherited Disease Research, was provided by the NIH GEI (U01HG004438), the National Institute on Alcohol Abuse and Alcoholism, the National Institute on Drug Abuse, and the NIH contract "High throughput genotyping for studying the genetic contributions to human disease" (HHSN268200782096C). Data were obtained from dbGaP (http://www.ncbi.nlm.nih.gov/sites/entrez?db=gap) for The Atherosclerosis Risk in Communities (ARIC) Study through accession number phs000090.v1.p1. The Atherosclerosis Risk in Communities Study is carried out as a collaborative study supported by National Heart, Lung, and Blood Institute contracts N01-HC-55015, N01-HC-55016, N01-HC-55018, N01-HC-55019, N01-HC-55020, N01-HC-55021, N01-HC-55022, R01HL087641, R01HL59367 and R01HL086694; National Human Genome Research Institute contract U01HG004402; and National Institutes of Health contract HHSN268200625226C. The authors thank the staff and participants of the ARIC study for their important contributions. Infrastructure was partly supported by Grant Number UL1RR025005, a component of the National Institutes of Health and NIH Roadmap for Medical Research. O.H is supported by the National Institute of Drug Abuse (DA027995) and the Barnes-Jewish Hospital Foundation.

The funders had no role in study design, data collection and analysis, decision to publish, or preparation of the manuscript.

