# Supplementary figures and images for: Pathway Analysis of Smoking Quantity in Multiple GWAS Identifies Cholinergic and Sensory Pathways
Source: PLoS One. 2012 Dec 5;7(12):e50913. doi: 10.1371/journal.pone.0050913 (PMC3515482; doi:10.1371/journal.pone.0050913)

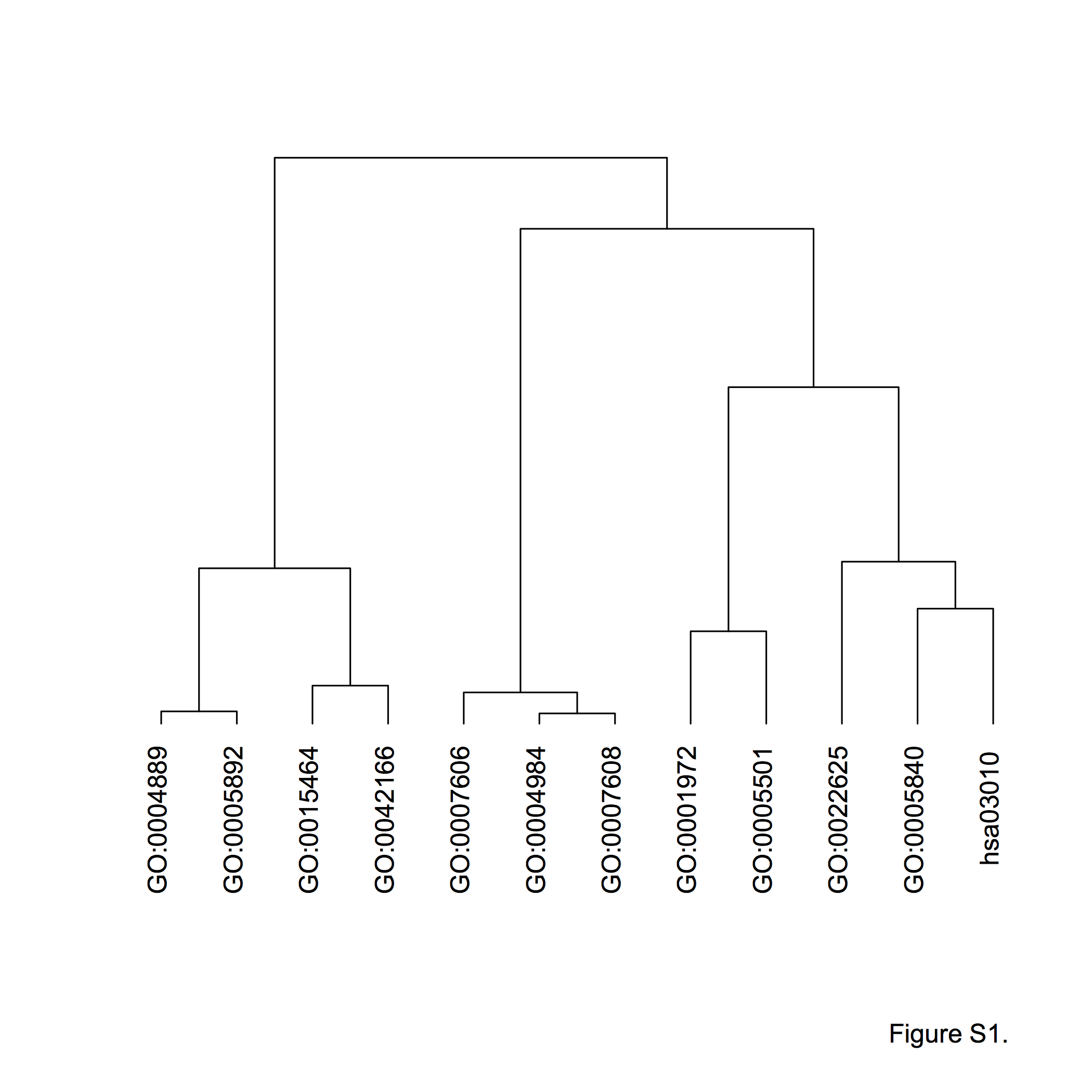

Supplement: Figure S1 — Hierarchical clustering of identified GO terms and KEGG pathway. We calculated the similarity matrix among the genes included in the 11 GO terms and KEGG pathway; and created a dendrogram by employing the single linkage method (i.e., nearest-neighbor). (TIFF) [file pone.0050913.s001.tiff]

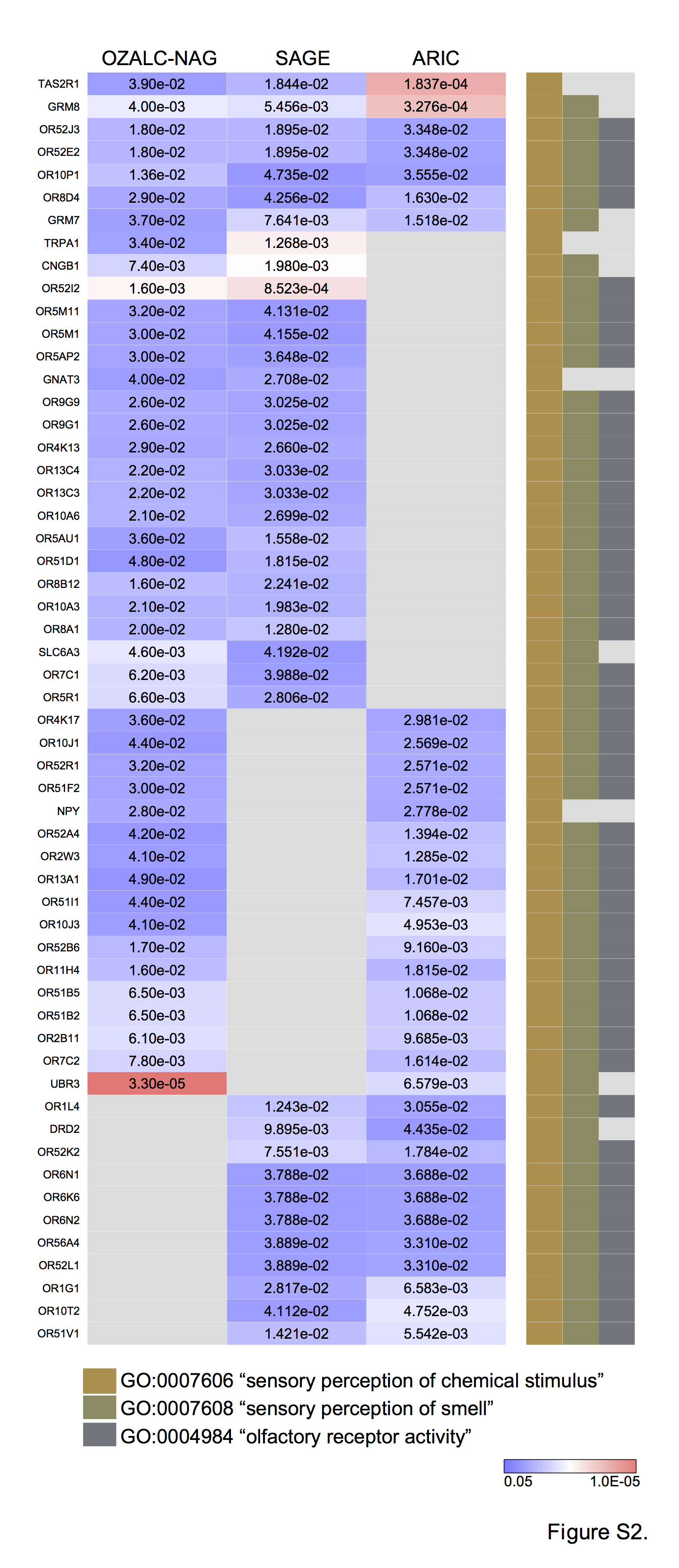

Supplement: Figure S2 — GO terms for the sensory perception of chemical stimulus and smell and significant genes. The p-value of each gene was assigned based on the most significant SNP in gene sequences and flanking regions (Left panel). SNPs in linkage disequilibrium (r2>0.2) and in a local proximity (1 Mb) were removed. Colored boxes in the right panel reflect the assignment of each gene to the different GO terms. Only genes significant in at least two studies are reported. (TIFF) [file pone.0050913.s002.tiff]

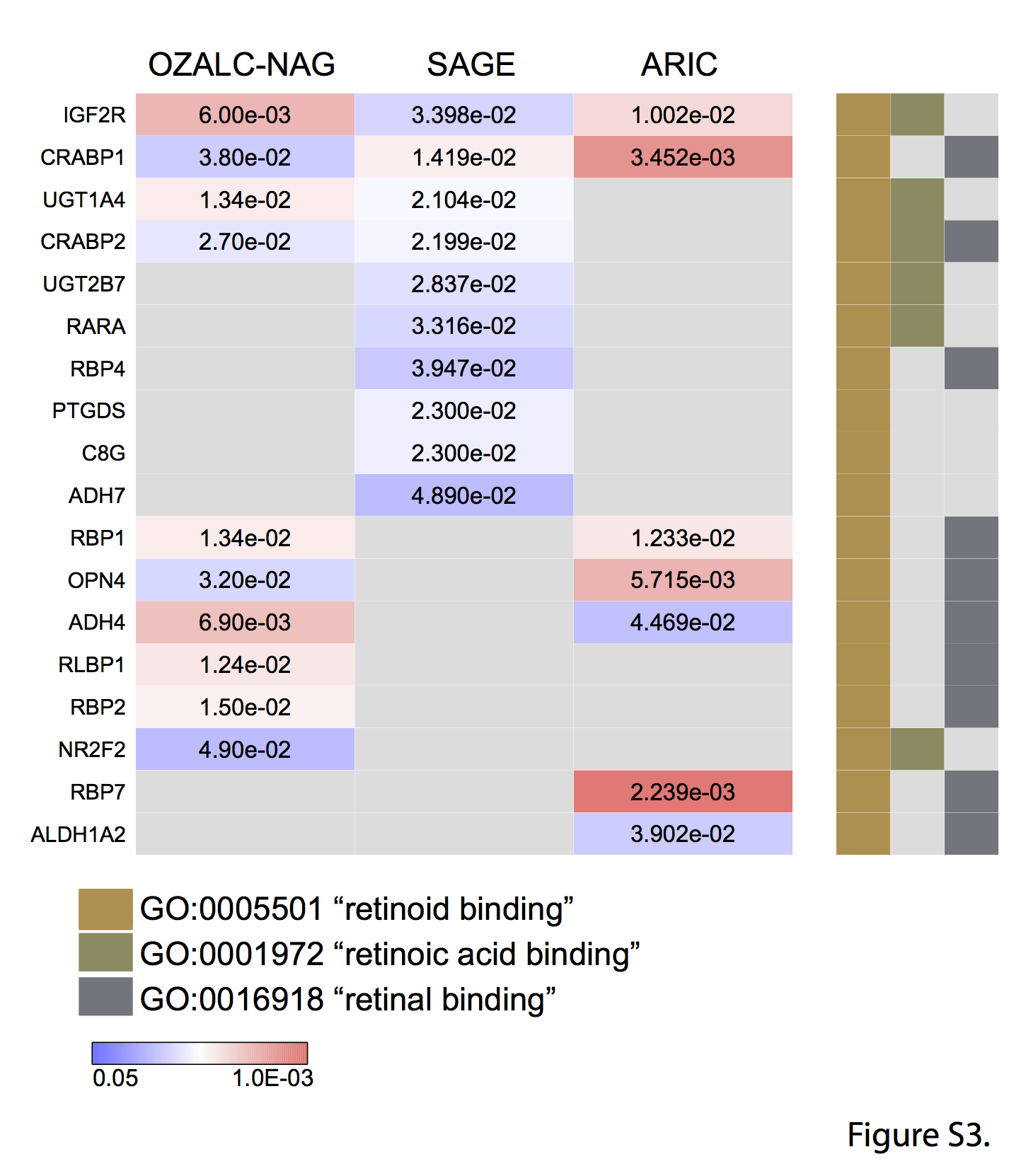

Supplement: Figure S3 — Significant genes for significant GO terms related to Retinoid binding terms. Similar to Figure S2, the p-value of genes significant in any of the three studies (OZALC-NAG, SAGE or ARIC) is reported. (TIFF) [file pone.0050913.s003.tiff]
