# Supplementary material for: Pathway Analysis of Smoking Quantity in Multiple GWAS Identifies Cholinergic and Sensory Pathways
Source: PLoS One. 2012 Dec 5;7(12):e50913. doi: 10.1371/journal.pone.0050913 (PMC3515482; doi:10.1371/journal.pone.0050913)
Supplement: Table S1 — Excess of enriched categories of genes identified by ALIGATOR for OZALC-NAG and SAGE studies for smoking quantity for Gene Ontoloty terms (A) and KEGG pathways (B). (PDF) [file pone.0050913.s004.pdf]

Table S1.

A)

SNP Threshold	Study	GO term enrichment threshold 0.005 #cat.	0.005 p-value	0.01 #cat.	0.01 p-value	0.05 #cat.	0.05 p-value
0.001	OZALC-NAG	23	1.20E-03	35	4.40E-03	101	1.02E-02
	SAGE	15	6.40E-03	34	2.20E-03	119	4.60E-03
0.005	OZALC-NAG	18	3.18E-02	26	9.02E-02	139	9.40E-02
	SAGE	11	1.37E-01	34	1.20E-01	179	2.44E-02
0.01	OZALC-NAG	19	8.84E-02	37	9.12E-02	163	1.62E-01
	SAGE	21	7.46E-02	41	8.74E-02	192	1.01E-01
0.05	OZALC-NAG	33	3.44E-02	62	4.60E-02	262	2.33E-01
	SAGE	42	1.80E-02	81	3.10E-02	313	5.78E-02

B)

SNP Threshold	Study	KEGG pathways enrichment threshold 0.005 #cat.	0.005 p-value	0.01 #cat.	0.01 p-value	0.05 #cat.	0.05 p-value
0.001	OZALC-NAG	1	2.80E-03	1	1.16E-02	1	1.76E-01
	SAGE	0	1.00E+00	1	7.80E-03	3	1.80E-03
0.005	OZALC-NAG	0	1.00E+00	1	7.00E-02	8	7.70E-02
	SAGE	0	1.00E+00	1	7.44E-02	2	5.54E-01
0.01	OZALC-NAG	0	1.00E+00	0	1.00E+00	12	4.62E-02
	SAGE	0	1.00E+00	0	1.00E+00	5	3.48E-01
0.05	OZALC-NAG	6	8.00E-04	10	1.20E-03	19	1.54E-02
	SAGE	4	3.20E-03	4	2.60E-02	13	8.40E-02
