# Supplementary material for: Pathway Analysis of Smoking Quantity in Multiple GWAS Identifies Cholinergic and Sensory Pathways
Source: PLoS One. 2012 Dec 5;7(12):e50913. doi: 10.1371/journal.pone.0050913 (PMC3515482; doi:10.1371/journal.pone.0050913)
Supplement: Table S2 — ALIGATOR identified common OZALC-NAG and SAGE GO terms for the analysis of SNPS p-value <0.001 and ARIC replication results. acc: Category of gene and corresponding name; # Genes Cat.: the total number of genes grouped by the category; # genes significant; and category specific p-value; and Expected number of genes for SAGE, OZALC-NAG and ARIC studies. *Combined p-values were calculated employing the weighted Z-score method {Stouffer:1949ua} (PDF) [file pone.0050913.s005.pdf]

Table S2

acc	name	# Genes Cat.	OZALC-NAG			SAGE			ARIC			Combined
			# Genes	*p-value*	Expected	# Genes	*p-value*	Expected	# Genes	*p-value*	Expected	*p-value**
GO:0035095	behavioral response to nicotine	7	3	2.00E-05	0.034	2	2.60E-04	0.028	3	2.00E-05	0.032	2.97E-10
GO:0004889	nicotinic acetylcholine-activated cation-selective channel activity	17	4	2.00E-05	0.114	2	4.20E-03	0.095	4	2.00E-05	0.000	1.33E-09
GO:0005892	nicotinic acetylcholine-gated receptor-channel complex	16	4	2.00E-05	0.114	2	4.20E-03	0.095	4	2.00E-05	0.000	1.33E-09
GO:0042166	acetylcholine binding	22	4	4.00E-05	0.125	2	5.56E-03	0.105	4	4.00E-05	0.000	5.99E-09
GO:0015464	acetylcholine receptor activity	18	4	4.00E-05	0.110	2	4.16E-03	0.093	3	2.80E-04	0.000	4.74E-08
GO:0035094	response to nicotine	20	3	2.16E-03	0.263	2	2.13E-02	0.225	3	1.82E-03	0.002	2.11E-05
GO:0007274	neuromuscular synaptic transmission	15	2	2.75E-02	0.256	2	2.10E-02	0.218	2	2.50E-02	0.025	2.56E-03
