# Supplementary material for: Pathway Analysis of Smoking Quantity in Multiple GWAS Identifies Cholinergic and Sensory Pathways
Source: PLoS One. 2012 Dec 5;7(12):e50913. doi: 10.1371/journal.pone.0050913 (PMC3515482; doi:10.1371/journal.pone.0050913)
Supplement: Table S3 — RefSNP (rs) numbers for the SNPs tagging the GO terms for the cholinergic receptor genes. (PDF) [file pone.0050913.s006.pdf]

Table S3

	OZALC-NAG	SAGE	ARIC
CHRNA5	rs16969968	rs16969968	rs569207
CHRNA3	rs16969968	rs16969968	rs569207
CHRNB4	rs6495309	rs6495309	rs8040868
CHRNB3	rs4950	rs1530848	rs4950
CHRNA6	rs16891604		rs7012713
CHRNA9	rs4861079	rs4469115	rs10021263
CHRNA7	rs904951	rs4779969	rs11858834
CHRNA2	rs6557999	rs9773817	
CHRM5	rs8035805		
CHRM3	rs2355228		
CHRNB2	rs1127313		
CHRM2	rs17506733		
CHRM4			rs12574668
CHRM1		rs12418496	rs12418496
CHRNA4		rs3787138	
CHRNG		rs733603	
CHRND		rs733603	
