# Supplementary material for: Pathway Analysis of Smoking Quantity in Multiple GWAS Identifies Cholinergic and Sensory Pathways
Source: PLoS One. 2012 Dec 5;7(12):e50913. doi: 10.1371/journal.pone.0050913 (PMC3515482; doi:10.1371/journal.pone.0050913)
Supplement: Table S4 — MAGENTA False discovery rate for categories of genes with nominal p-value<0.05 in the OZALC-NAG and SAGE studies and ARIC corresponding results. (PDF) [file pone.0050913.s007.pdf]

Table S4

Acc	Name	OZALC-NAG *q-value*	SAGE *q-value*	ARIC *q-value*
GO:0035095	behavioral response to nicotine	1.90E-01	9.89E-02	5.34E-02
GO:0060084	synaptic transmission involved in micturition	4.53E-01	8.14E-02	6.34E-02
GO:0006942	regulation of striated muscle contraction	5.15E-01	2.25E-01	1.00E+00
GO:0004889	nicotinic acetylcholine-activated cation-selective channel activity	1.00E+00	3.90E-01	6.03E-02
GO:0005892	nicotinic acetylcholine-gated receptor-channel complex	5.42E-01	3.51E-01	4.86E-02
GO:0015464	acetylcholine receptor activity	1.70E-01	7.23E-01	8.54E-02
GO:0005230	extracellular ligand-gated ion channel activity	5.09E-01	4.55E-01	3.86E-01
GO:0007271	synaptic transmission, cholinergic	4.77E-01	5.83E-01	3.23E-01
GO:0042060	wound healing	4.93E-01	4.53E-01	8.11E-01
GO:0006940	regulation of smooth muscle contraction	5.88E-01	4.90E-01	5.77E-01
GO:0005216	ion channel activity	6.65E-01	4.07E-01	6.97E-01
GO:0042552	myelination	5.96E-01	4.75E-01	9.04E-01
GO:0007257	activation of JUN kinase activity	6.30E-01	3.59E-01	8.35E-01
GO:0006548	histidine catabolic process	6.33E-01	4.00E-01	7.10E-01
GO:0034185	apolipoprotein binding	6.30E-01	4.21E-01	9.97E-01
GO:0007265	Ras protein signal transduction	7.52E-01	4.46E-01	1.00E+00
