# Supplementary material for: Pathway Analysis of Smoking Quantity in Multiple GWAS Identifies Cholinergic and Sensory Pathways
Source: PLoS One. 2012 Dec 5;7(12):e50913. doi: 10.1371/journal.pone.0050913 (PMC3515482; doi:10.1371/journal.pone.0050913)
Supplement: Table S5 — ALIGATOR enrichment analysis of the collapsed genes (physical distance <1 Mb) for GO terms representing the cholinergic receptor and the sensory perception genes. (PDF) [file pone.0050913.s008.pdf]

Table S5

acc	Category of gene	OZALC-NAG	SAGE	ARIC
GO:0042166	acetylcholine binding	1.00E-03	1.00E-03	1.00E-02
GO:0015464	acetylcholine receptor activity	5.00E-03	2.60E-03	6.20E-03
GO:0004889	nicotinic acetylcholine-activated cation-selective channel activity	4.00E-03	1.00E-03	2.90E-02
GO:0005892	nicotinic acetylcholine-gated receptor-channel complex	4.00E-03	1.00E-03	2.90E-02
GO:0004984	olfactory receptor activity	2.54E-02	1.34E-02	2.80E-03
GO:0007608	sensory perception of smell	1.14E-01	1.91E-01	2.58E-01
GO:0007606	sensory perception of chemical stimulus	9.20E-02	1.87E-01	1.33E-01
