# Supplementary material for: Pathway Analysis of Smoking Quantity in Multiple GWAS Identifies Cholinergic and Sensory Pathways
Source: PLoS One. 2012 Dec 5;7(12):e50913. doi: 10.1371/journal.pone.0050913 (PMC3515482; doi:10.1371/journal.pone.0050913)
Supplement: Text S1 — Samples and study design. (DOCX) [file pone.0050913.s009.docx]

**Pathway analysis of smoking quantity in multiple GWAS identifies cholinergic and sensory pathways**

Oscar Harari PhD^1^, Jen-Chyong Wang PhD^1^, Kathleen Bucholz PhD^1^, Howard J. Edenberg PhD^2^, Andrew Heath DPhil^1^, Nicholas G. Martin PhD^3^, Michele L. Pergadia PhD^1^, Grant Montgomery PhD^4^, Andrew Schrage MS^1^, Laura J. Bierut MD^1^, Pamela F. Madden PhD^1^,and Alison M. Goate DPhil^1,*^

^1^Department of Psychiatry, Washington University School of Medicine, St. Louis, Missouri, USA

^2^ Department of Biochemistry and Molecular Biology, School of Medicine, Indiana University, Indianapolis, Indiana

^3^ Genetic Epidemiology, Queensland Institute of Medical Research, Brisbane, Australia

^4^ Molecular Epidemology, Queensland Institute of Medical Research, Brisbane, Australia

Supporting Text S1 - Samples and study design

Study of Addiction: Genetics and Environment (SAGE). This dataset includes unrelated individuals of European American (69%) and African American (31%) descent. We analyzed the EA subjects, with reported CPD values, selected from three studies: the Collaborative Genetic Study of Nicotine Dependence (N=1063), the Collaborative Study in Genetics of Alcoholism (N=626), and the Family Study of Cocaine Dependence (N=325). The assessment was based on the Semi-Structured Assessment for the Genetics of Alcoholism harmonized across all three studies. The study design is described elsewhere: ^1^; and can be accessed in dbGaP (study accession phs000092.v1.p1). We analyzed the quantity smoked per day (question 4 of the Fagerstrom test for nicotine dependence (FTND) ^2^), for the subjects with European ancestry (Table 1). The Institutional Review Board at each contributing institution reviewed and approved the protocols for genetic studies under which all subjects were recruited. Subjects provided written informed consent for genetic studies and agreed to have their DNA and phenotypic information available to qualified investigators ^1^

**Nicotine Addiction Genetics (OZALC-NAG)** ^3^. This study includes participants enrolled at the Queensland Institute of Medical Research (QIMR) in Australia (Table 1) from a pool of families identified through diagnostic interview surveys of two cohorts of the Australian twin panel, which included spouses of the older of these two cohorts, for a total of about 12,500 families with information about smoking. The ancestry of the Australian samples is predominantly Anglo-Celtic or northern European (>90%). Index cases from these families, their full siblings, and parents were recruited for three coordinated studies: 1) the Nicotine Addiction Genetics (OZALC-NAG) Study, which ascertained heavy smoking index cases ^4^; 2) the Australian Alcohol Extreme Discordant and Concordant Sibship (OZALCX-EDAC) study, which ascertained index cases with a history of alcohol dependence or heavy drinking (operationalized as in ^5^); and 3) the Australian Alcohol Large Sibship (OZALC-BIGSIB) study, which ascertained large sibships, regardless of sibling phenotypic values ^4,^ ^3^ Telephone interview survey data included FTND and DSM-IV-based assessments of nicotine dependence, as well as measures of quantity and frequency of cigarette use. In this study, we use a measure of quantity of cigarettes smoked on average per day during the heaviest period of smoking. All data collection procedures were approved by institutional review boards at Washington University and the Queensland Institute of Medical Research. If the subject was an index case, permission was requested to contact other family members.

Atherosclerosis Risk in Communities study (ARIC). This is a multicenter population-based study designed to investigate the etiology of atherosclerosis in middle-aged adults ^6^, recruited from four U.S. communities. We used the average estimated number of cigarettes smoked per day across the entire time the subject smoked; using the values from the fourth question of the FTND (≤10; 11-20; 21-30; and >30). We selected only those subjects with European ancestry (Table 1). This study is part of the Gene Environment Association Studies initiative funded by the trans-NIH Genes, Environment, and Health Initiative (GEI). We analyzed the limited set of the data, accessed in dbGaP (Study Accession: phs000090.v1.p1). The study was approved by institutional review boards at each center, and all participants gave informed consent.

**References**

1. Bierut LJ, Agrawal A, Bucholz KK, Doheny KF, Laurie C, Pugh E, *et al.* A genome-wide association study of alcohol dependence. *Proc Natl Acad Sci USA* 2010 Mar. 16; **107**: 5082–5087.

2. Heatherton TF, Kozlowski LT, Frecker RC, Fagerstrom K-O. The Fagerstrom Test for Nicotine Dependence: a revision of the Fagerstrom Tolerance Questionnaire. *Addiction* 1991 Sep.; **86**: 1119–1127.

3. Heath AC, Whitfield JB, Martin NG, Pergadia ML, Goate AM, Lind PA, *et al.* A Quantitative-Trait Genome-Wide Association Study of Alcoholism Risk in the Community: Findings and Implications. *Biol Psychiatry* 2011 Apr. 27;

4. Saccone S. Genetic Linkage to Chromosome 22q12 for a Heavy-Smoking Quantitative Trait in Two Independent Samples. *The American Journal of Human Genetics* 2007 May; **80**: 856–866.

5. Grant JD, Agrawal A, Bucholz KK, Madden PAF, Pergadia ML, Nelson EC, *et al.* Alcohol consumption indices of genetic risk for alcohol dependence. *Biol Psychiatry* 2009 Oct. 15; **66**: 795–800.

6. Sharrett AR. The Atherosclerosis Risk in Communities (ARIC) Study. Introduction and objectives of the hemostasis component. *Ann Epidemiol* 1992 Jul.; **2**: 467–469.
