# Supplementary material for: Pathway Analysis of Smoking Quantity in Multiple GWAS Identifies Cholinergic and Sensory Pathways
Source: PLoS One. 2012 Dec 5;7(12):e50913. doi: 10.1371/journal.pone.0050913 (PMC3515482; doi:10.1371/journal.pone.0050913)
Supplement: Text S2 — Genotypes. (DOCX) [file pone.0050913.s010.docx]

**Pathway analysis of smoking quantity in multiple GWAS identifies cholinergic and sensory pathways**

Oscar Harari PhD^1^, Jen-Chyong Wang PhD^1^, Kathleen Bucholz PhD^1^, Howard J. Edenberg PhD^2^, Andrew Heath DPhil^1^, Nicholas G. Martin PhD^3^, Michele L. Pergadia PhD^1^, Grant Montgomery PhD^4^, Andrew Schrage MS^1^, Laura J. Bierut MD^1^, Pamela F. Madden PhD^1^,and Alison M. Goate DPhil^1,*^

^1^Department of Psychiatry, Washington University School of Medicine, St. Louis, Missouri, USA

^2^ Department of Biochemistry and Molecular Biology, School of Medicine, Indiana University, Indianapolis, Indiana

^3^ Genetic Epidemiology, Queensland Institute of Medical Research, Brisbane, Australia

^4^ Molecular Epidemology, Queensland Institute of Medical Research, Brisbane, Australia

Supporting Text S2 - Genotypes

SAGE. Genotyping was performed using Illumina Human1Mv1_C BeadChips. Data cleaning and quality-control standards are described in detail in ^1^. We analyzed 391,003 SNPs with a minor allele frequency (MAF) > 5% in populations of European descent, according to the human genome assembly build 37.1.

OZALC-NAG. Genotyping was conducted on Illumina platforms, including the Human 317K, the Human CNV370-Quadv3, and the Human 610-Quad. A detailed description of the genotypic data, data cleaning and quality-control measures is available in^2^. We analyzed the SNPs genotyped in at least 2000 subjects, and with a MAF > 5%. Employing the human genome assembly build 37.1, a total of 154,477 SNPs met these criteria. All of these SNPs were also present on the Illumina Human 1M platform. We extended the set of SNPs analyzed to match the ones evaluated on the Illumina Human 1M by including imputing SNPs. Imputation was performed using MaCH^3^ and HapMap samples of European ancestry (CEU; build 36, release 22) as the reference population^2^.

ARIC. Genotyping was performed using the Affymetrix Genome-Wide Human SNP Array 6.0. Data cleaning and quality-control standards are described elsewhere: ^4^. We analyzed 96,902 SNPs with a MAF > 5% in European descent populations that were also included in the Illumina Human1M. We extended this set of SNP including imputed SNPs to match the ones genotyped in the Illumina Human 1M chip. Imputation was performed at the GENEVA coordination center^5^, executing BEAGLE version 3.3^6^ on the full set of SNPs genotyped and employing HapMap Phase 3 samples of European ancestry (CEU + TSI) as the reference population^7^ .

**References**

1. Bierut LJ, Agrawal A, Bucholz KK, Doheny KF, Laurie C, Pugh E, *et al.* A genome-wide association study of alcohol dependence. *Proc Natl Acad Sci USA* 2010 Mar. 16; **107**: 5082–5087.

2. Medland SE, Nyholt DR, Painter JN, McEvoy BP, McRae AF, Zhu G, *et al.* Common variants in the trichohyalin gene are associated with straight hair in Europeans. *Am J Hum Genet* 2009 Nov.; **85**: 750–755.

3. Li Y, Willer CJ, Ding J, Scheet P, Abecasis GR. MaCH: using sequence and genotype data to estimate haplotypes and unobserved genotypes. *Genet Epidemiol* 2010 Dec.; **34**: 816–834.

4. Rasmussen-Torvik LJ, Alonso A, Li M, Kao W, Köttgen A, Yan Y, *et al.* Impact of repeated measures and sample selection on genome-wide association studies of fasting glucose. *Genet Epidemiol* 2010 Nov.; **34**: 665–673.

5. Cornelis MC, Agrawal A, Cole JW, Hansel NN, Barnes KC, Beaty TH, *et al.* The gene, environment association studies consortium (GENEVA): maximizing the knowledge obtained from GWAS by collaboration across studies of multiple conditions. *Genet Epidemiol* 2010 Jan. 20; **34**: 364–372.

6. Browning BL, Browning SR. A unified approach to genotype imputation and haplotype-phase inference for large data sets of trios and unrelated individuals. *Am J Hum Genet* 2009 Feb.; **84**: 210–223.

7. GENEVA Coordinating Center. GENEVA ARIC project imputation report [Internet]. 2010. Available from: https://www.genevastudy.org/sites/www/content/files/datacleaning/imputation/ARIC_Imputation_Report.pdf
