# Supplementary material for: Pathway Analysis of Smoking Quantity in Multiple GWAS Identifies Cholinergic and Sensory Pathways
Source: PLoS One. 2012 Dec 5;7(12):e50913. doi: 10.1371/journal.pone.0050913 (PMC3515482; doi:10.1371/journal.pone.0050913)
Supplement: Text S3 — Statistical analysis. (DOCX) [file pone.0050913.s011.docx]

**Pathway analysis of smoking quantity in multiple GWAS identifies cholinergic and sensory pathways**

Oscar Harari PhD^1^, Jen-Chyong Wang PhD^1^, Kathleen Bucholz PhD^1^, Howard J. Edenberg PhD^2^, Andrew Heath DPhil^1^, Nicholas G. Martin PhD^3^, Michele L. Pergadia PhD^1^, Grant Montgomery PhD^4^, Andrew Schrage MS^1^, Laura J. Bierut MD^1^, Pamela F. Madden PhD^1^,and Alison M. Goate DPhil^1,*^

^1^Department of Psychiatry, Washington University School of Medicine, St. Louis, Missouri, USA

^2^ Department of Biochemistry and Molecular Biology, School of Medicine, Indiana University, Indianapolis, Indiana

^3^ Genetic Epidemiology, Queensland Institute of Medical Research, Brisbane, Australia

^4^ Molecular Epidemology, Queensland Institute of Medical Research, Brisbane, Australia

Supporting Text S3 - Statistical analysis

Association p-values were calculated for unrelated samples datasets (SAGE and ARIC) employing linear regression models implemented in PLINK v1.07 ^1^, including gender as a covariate. In the case of the SAGE dataset, we also adjusted for alcohol and cocaine dependence and age, which was represented as quartiles (<35; 35-40; 40-45; and ≥45) ^2^. We employed MERLIN v1.1.2 ^3^ to perform the association test for the family-based OZALC-NAG dataset (fastAssoc option), adjusting for age and gender.

**References**

1. Purcell S, Neale B, Todd-Brown K, Thomas L, Ferreira MAR, Bender D, *et al.* PLINK: a tool set for whole-genome association and population-based linkage analyses. *Am J Hum Genet* 2007 Sep.; **81**: 559–575.

2. Bierut LJ, Agrawal A, Bucholz KK, Doheny KF, Laurie C, Pugh E, *et al.* A genome-wide association study of alcohol dependence. *Proc Natl Acad Sci USA* 2010 Mar. 16; **107**: 5082–5087.

3. Abecasis GR, Cherny SS, Cookson WO, Cardon LR. Merlin--rapid analysis of dense genetic maps using sparse gene flow trees. *Nat Genet* 2002 Jan.; **30**: 97–101.
